# Thrombocytopenia and Postpartum Hemorrhage in a Woman with Chromosome 22q11.2 Deletion Syndrome

**DOI:** 10.1155/2016/2920375

**Published:** 2016-06-05

**Authors:** Sarah L. Pachtman, Kathy Deng, Deepak Nanda

**Affiliations:** ^1^Division of Maternal Fetal Medicine, Department of Obstetrics and Gynecology, Hofstra Northwell School of Medicine, Long Island Jewish Medical Center, New Hyde Park, NY 11042, USA; ^2^Division of Hematology-Oncology, Department of Internal Medicine, Hofstra Northwell School of Medicine, Long Island Jewish Medical Center, New Hyde Park, NY 11042, USA

## Abstract

Chromosome 22q11.2 deletion syndrome, also known as DiGeorge or velocardiofacial syndrome, is associated with a wide spectrum of phenotypic features. It is known to be associated with severe macrothrombocytopenia. Postpartum hemorrhage is a leading cause of maternal morbidity and mortality globally. Chromosome 22q11.2 deletion syndrome is rare cause of thrombocytopenia that can be a significant risk factor for life-threatening postpartum hemorrhage. We report a case of postpartum hemorrhage in a woman with 22q11.2 deletion syndrome causing severe macrothrombocytopenia.

## 1. Introduction

Chromosome 22q11.2 deletion syndrome, also known as DiGeorge or velocardiofacial syndrome, is the most common microdeletion syndrome and is associated with a wide spectrum of phenotypic features. The estimated incidence is 1 in 2,000–6,000 live births [1]. It is inherited in an autosomal dominant pattern and clinical findings can vary even among affected family members. Some of the most common characteristics include cardiac anomalies, facial dysmorphism, developmental delay, autoimmune disorders, and velopharyngeal insufficiency [2]. It is also known to be associated with thrombocytopenia [3] and recurrent epistaxis in up to 12% of patients [4].

Postpartum hemorrhage is a leading cause of maternal mortality and of near-miss cases that can result in significant morbidity [5]. Severe maternal thrombocytopenia, though a rare cause of bleeding at time of delivery, can be a risk factor for maternal hemorrhage [6].

## 2. Case

A 24-year-old Mexican female, G3P0020, with a singleton intrauterine pregnancy presented as a transfer of care from outside facility secondary to fetal cardiac anomaly noted on comprehensive sonogram. Fetal echocardiogram revealed tetralogy of Fallot with pulmonary atresia. Amniocentesis cell culture resulted normal 46, XX karyotype and chromosomal microarray analysis detected an interstitial deletion of chromosome 22q11.2.

At 37 weeks and 4 days of gestational age, she presented with epistaxis and was found to have decreased platelet count of 40 K/*μ*L. Her baseline platelet count was 150 K/*μ*L in the first trimester. At that time, the plan was to start oral prednisone for presumed ITP during pregnancy and to monitor weekly platelet count. However, during the appointment, she developed brisk and massive epistaxis and was promptly admitted to the hospital for observation and therapy for thrombocytopenia.

When interviewed, she reported no significant past medical history and no personal or family history of coagulation disorders or congenital cardiac anomalies. She denied learning difficulties or developmental delay. Her prenatal course was significant for a history of vulvar herpes simplex with no current active lesions. Her only medications were prenatal vitamins and prophylactic oral valacyclovir. She did not have abnormal facies or apparent developmental delay. No petechia or ecchymosis was noted on skin exam and she was neurologically intact. Serum hemoglobin measured 10.2 g/dL. Large platelets were seen on peripheral smear and coagulation profile was normal. Maternal transthoracic echocardiogram was normal.

During her antepartum course, she had minimal response to oral prednisone therapy, so two-day course of intravenous immunoglobulin (IVIG) was started. Hemoglobin measured 8.9 g/dL and she was treated with oral ferrous sulfate, vitamin B12, and folic acid. Epistaxis was adequately controlled with nasal saline with plan for nasal oxymetazoline and pressure in the case of intractable bleeding. After the course of IVIG, platelet count was only 27 K/*μ*L, so trial of pulse dexamethasone therapy was initiated. Dexamethasone therapy continued for four days with no response in platelet count, and she was transitioned back to oral prednisone therapy. She was transfused one unit of platelets during her antepartum course once platelet count reached a nadir of 15 K/*μ*L, as she was not actively bleeding and there was concern for consumption of transfused platelets by autoantibodies. Coagulation profile remained normal. She had a minimal response to receiving platelets, with posttransfusion platelet count of 27 K/*μ*L.

At 39 weeks and 3 days of gestational age, she went into spontaneous labor. During her labor course, her blood pressure became elevated into the severe range, requiring antihypertensive agents for control. She reported a headache, and magnesium sulfate infusion was initiated for seizure prophylaxis. Platelet transfusion of half units over three hours every 12 hours was started once cervical exam was 6 cm. Repeat platelet count at this time was 25 K/*μ*L with a goal of 50 K/*μ*L in case of need for cesarean delivery. Her labor was protracted in the active phase, requiring augmentation with oxytocin. Intravenous analgesia with narcotics was used for pain control.

She delivered a 3460 g viable female infant vaginally over second-degree perineal laceration. Magnesium sulfate infusion was discontinued once fetus was at +3 station. Apgar scores were 8 at 1 minute and 9 at 5 minutes. The neonate was immediately transferred to neonatal intensive care unit for care. More aggressive transfusion of platelets was initiated at delivery. During labor and delivery course, she received a total of 7 units of transfused platelets with a response to 146 K/*μ*L.

The placenta did not spontaneously separate after thirty minutes and was removed manually. Immediately following delivery, she began to bleed from vaginal laceration repair site and lower uterine segment with no resolution after misoprostol and carboprost administration. She was noted to have epistaxis and bleeding from oral mucous membranes. An intrauterine balloon tamponade device was placed and uterine bleeding successfully controlled. At this time, transfusion of platelets continued and transfusion of two units of packed red blood cells and two units of fresh frozen plasma was initiated. She began to have labored breathing and inability to maintain oxygen saturation and was intubated and transferred to medical intensive care unit for management of presumed transfusion associated cardiac overload. Chest radiograph noted severe pulmonary edema. Seizure prophylaxis was continued with levetiracetam. Successful diuresis allowed extubation after 24 hours, and the remainder of her postpartum hospitalization was uncomplicated.

During her postpartum course, she was continued on intravenous steroid therapy and was able to maintain platelet counts above 80 K/*μ*L. She was discharged with a platelet count of 98 K/*μ*L. Bone marrow biopsy was performed, final pathology revealed normocellularity (50 to 55% cellularity), trilineage hematopoiesis with maturation, normal M : E ratio, megakaryocytes normal in number and morphology, and mild perivascular plasmacytosis, and iron stores were present. Maturing and mature myeloid and erythroid elements were present with normal M : E ratio (2.7 : 1). Megakaryocytes appeared slightly increased in number with normal morphology. These findings are consistent with an immune etiology of macrothrombocytopenia.

Antiphospholipid antibodies were normal when tested postpartum. Maternal peripheral blood cytogenetics and fluorescence in situ hybridization (FISH) performed at this time revealed a deletion in chromosome 22q11.2. Four months postpartum, platelet count was 104 K/*μ*L, and she had no further bleeding complications. At this time, she presented with a complaint of flank pain, and a renal sonogram was performed that also documented a normal sized spleen. Neonate had normal platelet counts and underwent successful ventricular septal defect closure and corrective repair of tetralogy of Fallot at nine months of age.

## 3. Discussion

Immune cytopenias and macrothrombocytopenia are known findings in chromosome 22q11.2 deletion syndrome and are thought to be about 200 times more prevalent than in the general population [7]. Thrombocytopenia can occur in up to 35% of patients with 22q11.2 deletion and large platelets can occur in up to 82% [8]. Thrombocytopenia has not been correlated with cardiac anomalies or other common phenotypic findings [9]. The etiology has been described as autoimmune; however, in some cases no clear etiology is identified [3]. The mechanism of autoimmunity is unknown [10].

Hypersplenism is also thought to be the cause of thrombocytopenia; however in her case, her spleen size was normal. This finding does not rule out a diagnosis of immune thrombocytopenic purpura (ITP). Some reports conclude that thrombocytopenia is associated with prolonged bleeding time and is thought to be secondary haploinsufficiency of the GP1b gene, which is found within the 22q11-deleted region [3, 11]. The GP1b gene encodes a platelet receptor that is imperative for platelet adhesion and thrombin formation. In other reports, patients were not found to have abnormal prothrombin time, thrombin time, partial thromboplastin time, bleeding time, or fibrinogen levels [9]. However, the degree of thrombocytopenia is the biggest predictor for the risk of hemorrhage [9, 12].

Thrombocytopenia is also a common complication in pregnancy. Gestational and immune thrombocytopenias are the most common etiologies. Generally the platelet count in gestational thrombocytopenia does not fall below 70 K/*μ*L and is not associated with significant bleeding risk [13]. Management of ITP includes treatment with steroids and intravenous immunoglobulin if there is an indication for rapid increase in platelet count. Careful monitoring of platelet counts is important for safety of regional anesthesia during labor, and vaginal and cesarean deliveries are generally thought to be safe with counts >50 K/*μ*L [6]. Other etiologies include preeclampsia and HELLP syndrome and thrombotic thrombocytopenic purpura (TTP). These diagnoses must also be considered and ruled out in a pregnant patient. Other immune causes of thrombocytopenia such as antiphospholipid antibody syndrome should also be investigated.

A diagnosis of chromosome 22q11.2 deletion syndrome should be considered in patients with severe or recurrent immune thrombocytopenias [14, 15], even in patients without or with mild expression of other features. Thrombocytopenia has been evaluated as a diagnostic predictor of 22q11.2 deletion syndrome, and although it has poor sensitivity and specificity as a diagnostic marker, this is secondary to the very broad spectrum of associated phenotypes [1]. In our patient, severe macrothrombocytopenia was the most pronounced phenotype and was only discovered during her pregnancy. Further coagulopathy is often a result of postpartum hemorrhage as clotting factors and platelets are consumed in the process, making severe thrombocytopenia a more dangerous risk factor. In addition, evaluation of the infant and communication with pediatricians are important, as neonatal thrombocytopenia can result from immune mechanisms. Chromosome 22q11.2 deletion syndrome is a rare cause of macrothrombocytopenia and platelet dysfunction that can pose significant risk for life-threatening postpartum hemorrhage.
